# Monkeypox Virus Transmission to Healthcare Worker through Needlestick Injury, Brazil

**DOI:** 10.3201/eid2811.221323

**Published:** 2022-11

**Authors:** Laína Bubach Carvalho, Luciana V.B. Casadio, Matheus Polly, Ana Catharina Nastri, Anna Cláudia Turdo, Raissa H. de Araujo Eliodoro, Ester Cerdeira Sabino, Anna Sara Levin, Adriana Coracini Tonacio de Proença, Hermes Ryoiti Higashino

**Affiliations:** Universidade de São Paulo Hospital das Clínicas, São Paulo, Brazil (L. Bubach Carvalho, L.V.B. Casadio, M. Polly, A.C. Nastri, A.C. Tonacio de Proença, H.R. Higashino);; Sao Camilo Hospital Pompeia Unit, São Paulo (L. Bubach Carvalho, L.V.B. Casadio, M. Polly, A.C. Turdo, A.C. Tonacio de Proença, H.R. Higashino);; Universidade de São Paulo Instituto de Medicina Tropical, São Paulo (R.H. de Araujo Eliodoro);; Universidade de São Paulo, São Paulo (E.C. Sabino, A.S. Levin)

**Keywords:** monkeypox, viruses, monkeypox virus, zoonoses, healthcare-associated infections, needlestick injuries, occupational exposure, nosocomial infections, Brazil

## Abstract

We describe monkeypox virus (MPXV) transmission from a patient to a healthcare worker through needlestick injury. A lesion appeared at the inoculation site 5 days after injury. Blood tested MPXV-positive by PCR before symptoms worsened; blood remained MPXV-positive at discharge 19 days after symptom onset. Postexposure prophylaxis could prevent potential MPXV bloodborne transmission.

In July 2022, the World Health Organization declared the global monkeypox outbreak a public health emergency (*1*). Monkeypox virus (MPXV) is transmitted through close or direct contact with skin lesions or respiratory droplets and through fomites, but knowledge gaps about transmission persist. 

During the ongoing outbreak, MPX has disproportionately affected men who have sex with men, suggesting amplification through sexual networks (*2*). MPXV transmission to healthcare workers (HCWs) in endemic settings is well described (*3*) but has not been well characterized in the current outbreak. In nonendemic countries, monkeypox is rare, and standard infection control precautions are applied, suggesting HCWs are at low risk of acquiring MPXV; only 1 prior HCW case has been reported (*4*). We describe MPXV transmission to a HCW in Brazil through a needlestick injury.

On July 9, 2022, a female nurse in her 20s sustained a needlestick injury to her thumb from supplies used to collect cutaneous lesion samples from a monkeypox patient. The nurse was wearing personal protective equipment, including gown, gloves, goggles, and mask, and was gathering materials to discard in a sharps container when a needle perforated her glove; the puncture site was visible immediately. After 5 days, a nodule developed at the injury site (day 0 of symptoms); it later evolved into a painful vesicle (Figure). The nurse lived alone, denied recent travel, and reported having protected sexual intercourse only with her male partner. She had no other potential exposures. 

**Figure:**
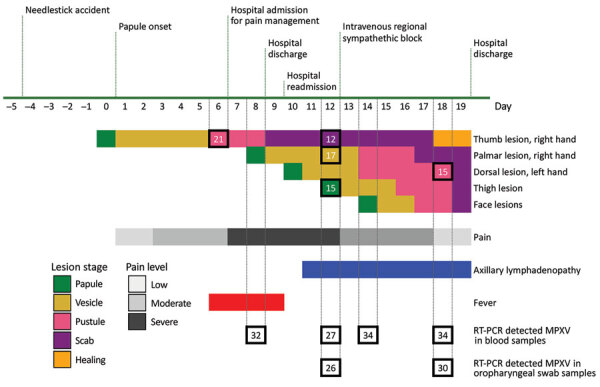
Timeline of symptoms and testing in a case of MPXV transmission to healthcare worker through needlestick injury, Brazil. All collected specimens had RT-PCR detectable MPXV through hospital discharge. Numerals inside squares indicate RT-PCR cycle threshold values. MPXV, monkeypox virus; RT-PCR, reverse transcription PCR.

The source patient, a man in his 20s who reported having sex with men, had mild monkeypox that started 2 weeks before the needlestick incident. He had sore throat, cervical lymphadenopathy, and sparse lesions on his face, torso, and groin. The patient and nurse provided written consent for this report.

 Overall, the nurse had 7 lesions: 1 each on the thumb (inoculation site) and palm of the right hand, dorsal left hand, and left thigh, and 3 on her face (Appendix Figures 1–3). Magnetic resonance imaging of the injury site on day 15 showed a neurovascular bundle and subcutaneous inflammation.

During the nurse’s follow-up, blood and skin lesion samples tested MPXV-positive by reverse transcription PCR using the QIAamp Viral DNA Mini Kit (QIAGEN, https://www.qiagen.com) for DNA extraction and TaqMan Monkeypox Virus Microbe Detection Assay (Thermo Fisher Scientific, https://www.thermofisher.com) for amplification. MPXV also was detectable in oropharyngeal samples despite the absence of respiratory symptoms. Of note, all collected specimens had detectable MPXV DNA throughout hospitalization. The nurse was discharged to outpatient care before complete lesion resolution (Figure).

In nonendemic settings, needlestick injury is an unusual form of patient-to-HCW MPXV transmission. Before 2022, fewer human-to-human than animal-to-human MPXV transmission cases were reported during outbreaks in Africa (*5*). In nonendemic countries, sporadic zoonotic or travel-associated monkeypox outbreaks have occurred (*5*,*6*), but during May–September 2022, >50,000 cases were reported worldwide (https://www.cdc.gov/poxvirus/monkeypox/response/2022/world-map.html), mainly through sexual or intimate contact transmission (*7*). HCWs are at risk, but a recent review of MPXV transmission in healthcare facilities in nonendemic countries found only 1 documented case of nosocomial monkeypox in a HCW, probably through contact with contaminated bedding (*4*,*8*).

Our case enabled observation of the natural progression of monkeypox through longitudinal clinical and laboratory monitoring of disease stages. The incubation period was 5 days. A cutaneous lesion and pain and inflammation at the inoculation site preceded generalized symptoms of fever and lymphadenopathy. The transmission route might have influenced the absence of a prodromal phase in the nurse because needlestick transmission parallels bite or scratch transmission from MPXV-infected animals to humans; in those cases, a febrile prodrome is uncommon (*5*). In addition, the nurse experienced severe injury site pain, which coincides with a series of cases in the current outbreak in which most patients who acquired MPXV by sexual or intimate contact were hospitalized for severe anorectal pain (*2*). The pain similarity suggests that the primary MPXV inoculation site is associated with painful lesions and possible neural impairment, as implied by the nurse’s magnetic resonance images. 

MPXV DNA detected in the nurse’s blood on day 8, before skin lesions appeared at distant sites, suggests hematogenous virus dissemination. Few reports describe MPXV DNA in blood, but a retrospective study of monkeypox antiviral treatment found detectable MPXV DNA in blood after 14 days, even after skin lesions resolved (*8*). How detectable MPXV DNA corresponds to true viremia is unknown, but persistent DNA suggests bloodborne transmission could be possible through needlesticks, blood transfusions, and organ transplants. Persistent MPXV DNA in the nurse’s oropharyngeal samples aligns with another report (*9*), but efficiency for droplet or airborne transmission remains unknown.

Because few documented needlestick monkeypox cases are available (*9*), we could not estimate transmission risk, but instruments used on cutaneous lesions likely pose a high risk. The World Health Organization recommends postexposure prophylaxis with second- or third-generation vaccine, if available, up to 4 days after exposure (*10*). The state of São Paulo, Brazil, discontinued smallpox vaccination after 1979, and no smallpox or monkeypox vaccine is available in Brazil. However, HCWs should be considered for vaccination as soon as it is available.

Our report describes clinical features of monkeypox, including extreme pain at the inoculation site and prolonged DNAemia, after needlestick transmission in a HCW. Preexposure and postexposure prophylaxis, including vaccination, should be provided for HCWs in Brazil.

Appendix Additional information on monkeypox virus transmission to healthcare worker through needlestick injury, Brazil.
