# Supplementary material for: Monkeypox Virus Transmission to Healthcare Worker through Needlestick Injury, Brazil
Source: Emerg Infect Dis. 2022 Nov;28(11):2334–6. doi: 10.3201/eid2811.221323 (PMC9622260; doi:10.3201/eid2811.221323)
Supplement: Appendix — Additional information on monkeypox virus transmission to healthcare worker through needlestick injury, Brazil. [file 22-1323-Techapp-s1.pdf]

Article DOI: https://doi.org/10.3201/eid2811.221323

Monkeypox Virus Transmission to Healthcare Worker through Needlestick Injury, Brazil

Appendix

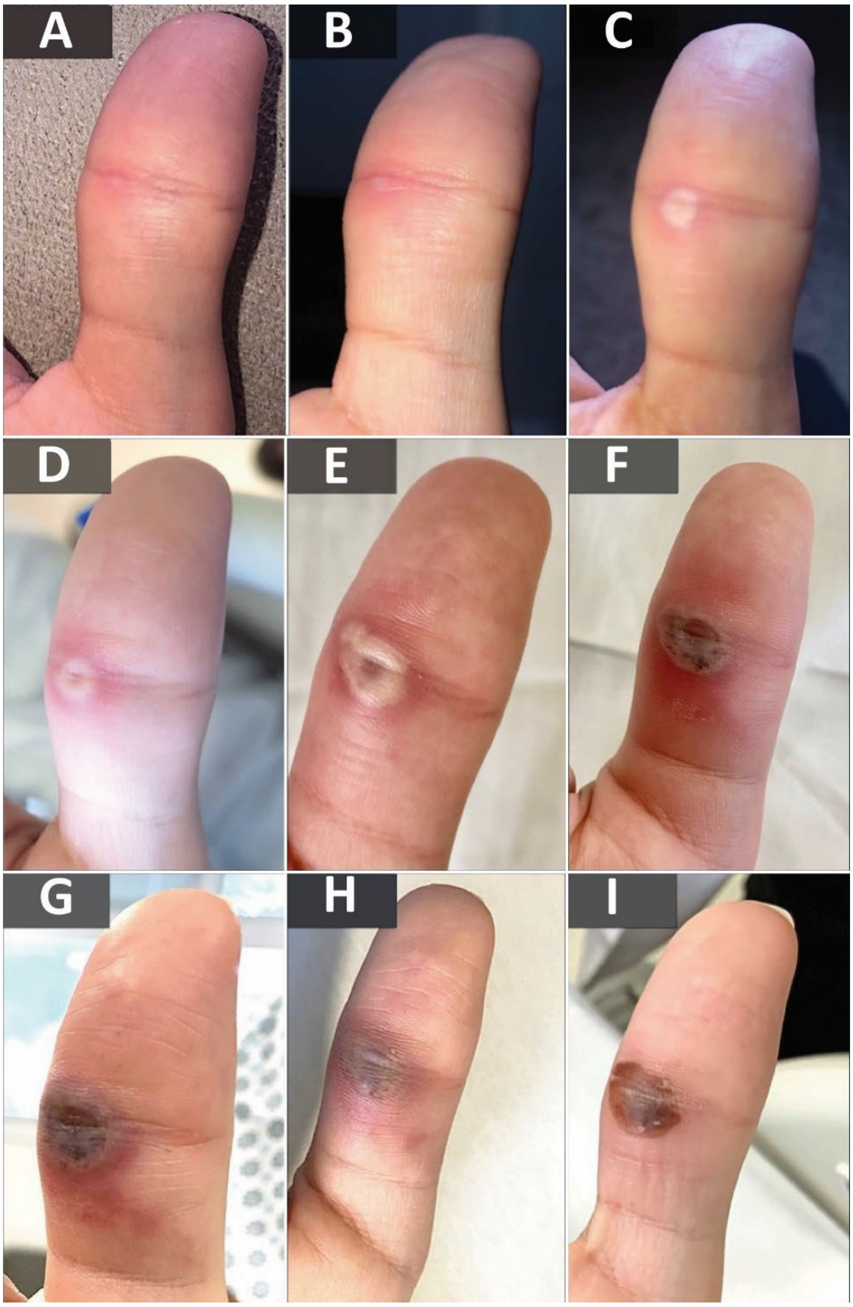

Appendix Figure 1. Site of needlestick in a case of monkeypox virus transmission to healthcare worker through needlestick injury, Brazil. Images show right thumb of the healthcare worker on day 0 (A), day 1 (B), day 4 (C), day 6 (D), day 8 (E), day 10 (F), day 12 (G), day 14 (H), and day 18 (I). Panel 2: New skin lesions (black arrows) and their development. Figures J, K, L show the same lesion over time. Figures M, N, O show the same lesion over time. Figures P and Q indicate new lesions on the thigh and the face, respectively.

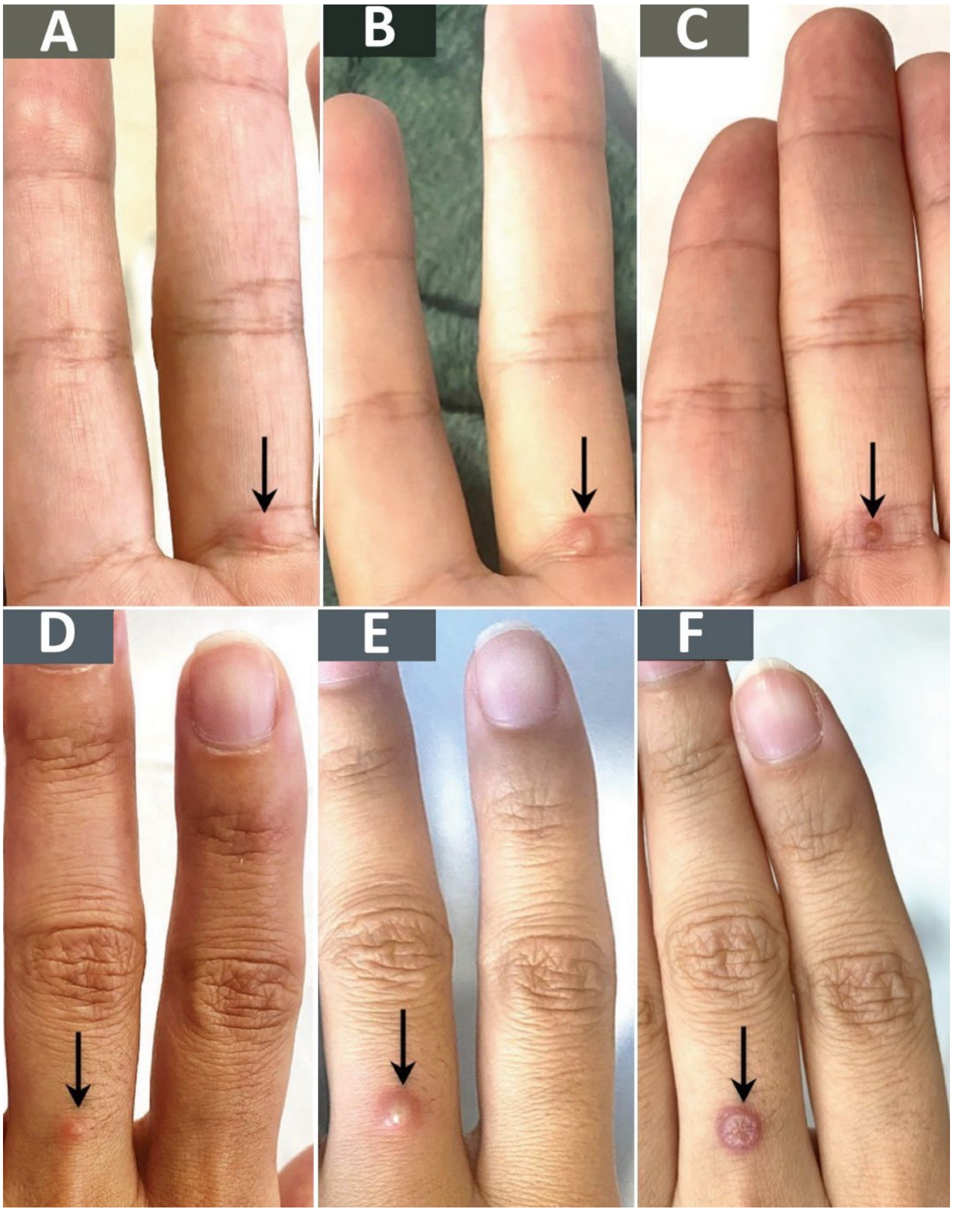

Appendix Figure 2. Additional skin lesions in a case of monkeypox virus transmission to healthcare worker through needlestick injury, Brazil. Black arrows indicate lesions on the palmar base of middle finger on the right hand (A–C) and on dorsal middle finger of left hand (D–F) over time: A) day 8; B,E) day 14; C,F) day 18; and D) day 10.

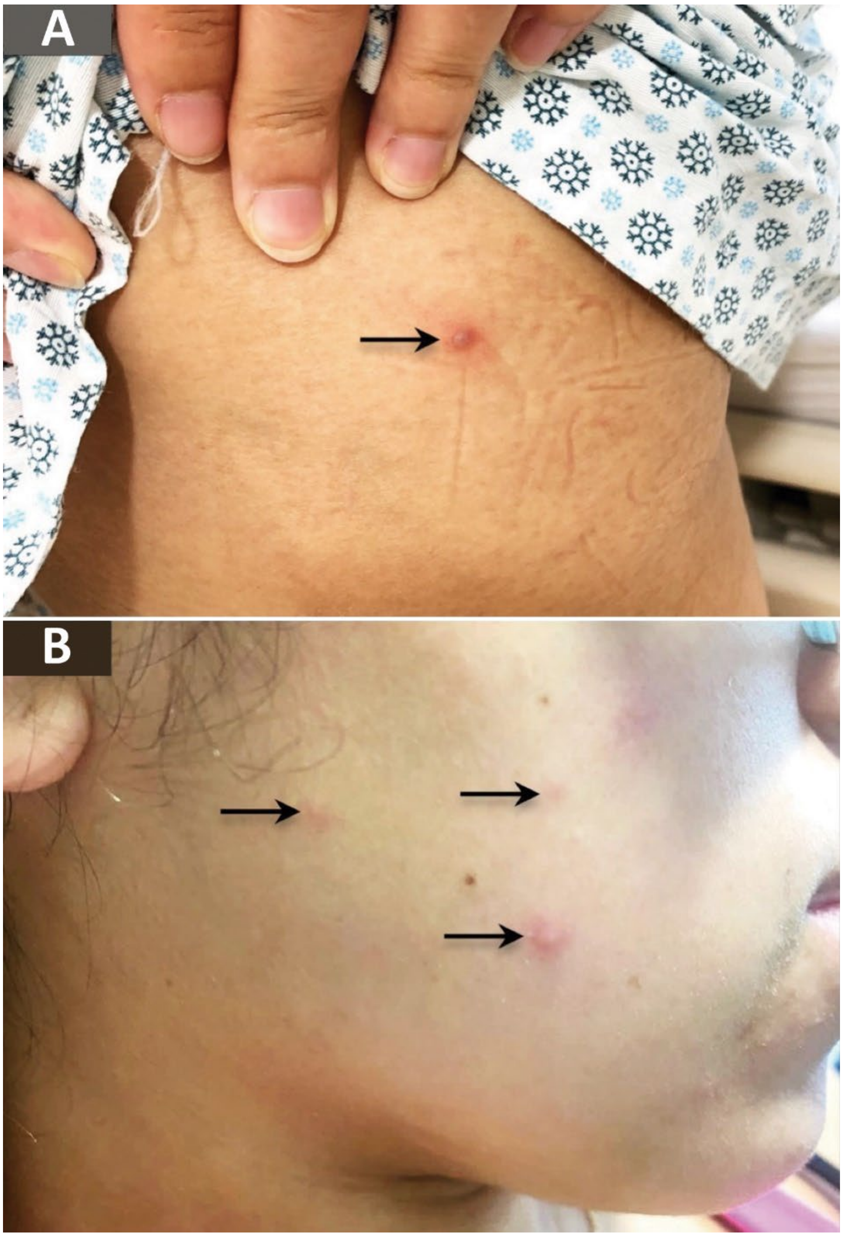

Appendix Figure 3. Additional skin lesions in a case of monkeypox virus transmission to healthcare worker through needlestick injury, Brazil. A) Thigh lesion; B) facial lesions.
